# Supplementary material for: Midbrain Tet1 dosage defines inter-individual binge-eating susceptibility
Source: bioRxiv. 2026 Mar 19:2026.03.14.711800. Preprint. [Version 2] doi: 10.64898/2026.03.14.711800 (PMC13015413; doi:10.64898/2026.03.14.711800)
Supplement: Supplementary file 1 [file NIHPP2026.03.14.711800v2-supplement-1.pdf]

bioRxiv preprint doi: https://doi.org/10.64898/2026.03.14.711800; this version posted March 19, 2026. The copyright holder for this preprint (which was not certified by peer review) is the author/funder, who has granted bioRxiv a license to display the preprint in perpetuity. It is made available under aCC-BY 4.0 International license.

Supplementary Material

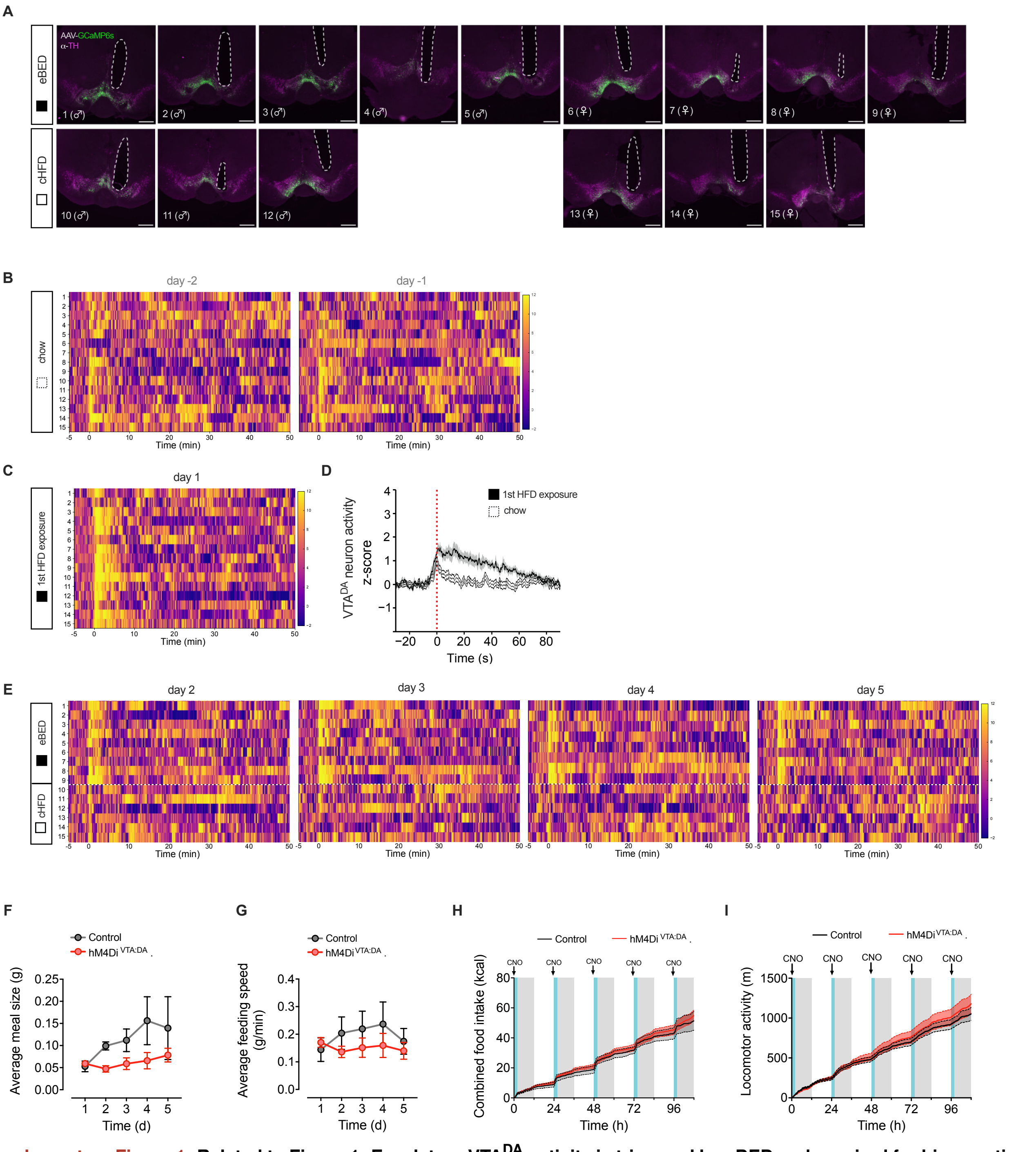

Supplementary Figure 1. Related to Figure 1. Escalatory VTA^{DA} activity is triggered by eBED and required for binge eating in mice. (A) Targeting validation of fiber photometry experiment including all mice; GCaMP6s expression colocalizing with VTA^{DA} neurons (TH^{+}) and optic fiber tract, respectively. **(B)** Heatmap representation of fiber photometry z-scores of individual mice over the course of the entire recording sessions on day –2 and day -1 (chow diet; food given at $T = 0$ min). $n = 6$–8 mice. **(C)** Heatmap representation of fiber photometry z-scores of individual mice over the course of the entire recording sessions on day 1 (first HFD exposure; food given at $T = 0$ min). $n = 6$–8 mice. **(D)** Fiber photometry VTA^{DA} recordings of mean z-score of the first HFD exposure versus the two baseline recordings (chow diet) at eating onset (0 min). Data are presented as mean $\pm$ SEM. $n = 6$–8 mice. **(E)** Heatmap representation of fiber photometry z-scores of individual mice over the course of the entire recording sessions on day 2–5 (eBED versus cHFD; food given at $T = 0$ min). $n = 6$–8 mice. **(F)** Average meal size per day of hM4Di$^{VTA:DA}$ mice relative to control mice. Data are presented as mean $\pm$ SEM. $n = 4$–7 mice. **(G)** Average eating speed per day of hM4Di$^{VTA:DA}$ mice relative to control mice. Data are presented as mean $\pm$ SEM. $n = 4$–7 mice. **(H)** Cumulative combined food intake (HFD + chow) of hM4Di$^{VTA:DA}$ mice relative to control mice. Time window with limited HFD access (shaded) and CNO injection (1 mg/kg BW; i.p.; arrow) are indicated. Data are presented as mean $\pm$ SEM. $n = 4$–7 mice. **(I)** Cumulative locomotor activity of hM4Di$^{VTA:DA}$ mice relative to control mice. Time window with limited HFD access (shaded) and CNO injection (1 mg/kg BW; i.p.; arrow) are indicated. Data are presented as mean $\pm$ SEM. $n = 4$–7 mice.

bioRxiv preprint doi: https://doi.org/10.64898/2026.03.14.711800; this version posted March 19, 2026. The copyright holder for this preprint (which was not certified by peer review) is the author/funder, who has granted bioRxiv a license to display the preprint in perpetuity. It is made available under aCC-BY 4.0 International license.

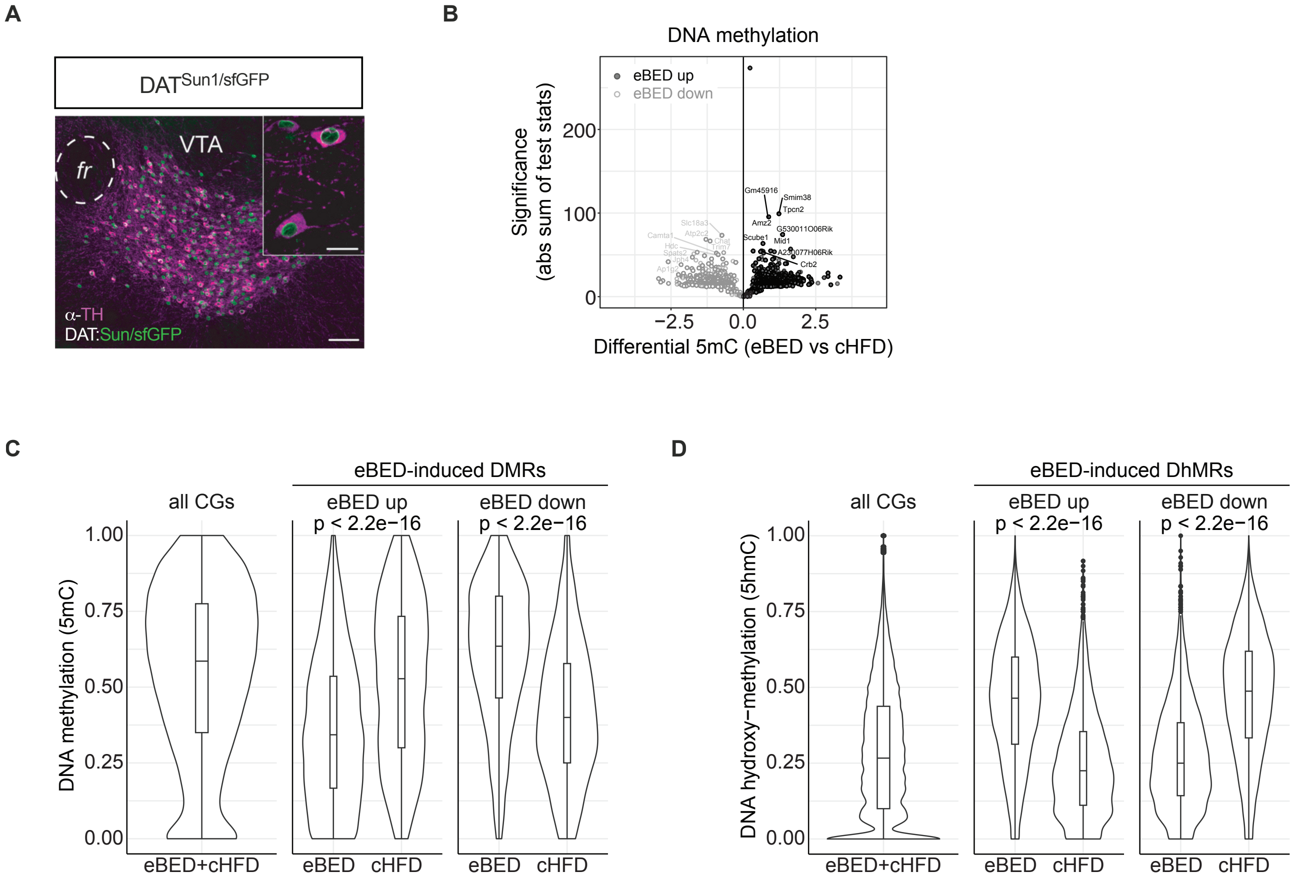

Supplementary Figure 2. Related to Figure 2. eBED triggers rapid and profound rewiring of the VTA^{DA} neuron epigenome. **(A)** Confocal micrograph of DAT$^{Sun1/sfGFP}$ reporter mice with GFP-tagged VTA^{DA} nuclei; scale bars: 50 µm, 20 µm (insert). **(B)** Volcano plot of DMRs comparing eBED versus cHFD. **(C)** Violin plots showing the levels of 5mC on all eBED+cHFD CGs (reference) and eBED-induced DMRs, showing decreased (left) or increased (right) amount of the DNA mark with respect to cHFD. P-values from Wilcoxon test. **(D)** Violin plots showing the levels of 5hmC on all eBED+cHFD CGs (reference) and eBED-induced DhMRs, showing increased (left) or decreased (right) amount of the DNA mark with respect to cHFD. P-values from Wilcoxon test.

In all panels, DMRs' effect size cut-off = 0.05; p-value cut-off = 0.01 from Wald statistical testing. $N = 12$ (2 samples each condition — eBED, cHFD, chow — and each mark -5mC, 5hmC).

bioRxiv preprint doi: https://doi.org/10.64898/2026.03.14.711800; this version posted March 19, 2026. The copyright holder for this preprint (which was not certified by peer review) is the author/funder, who has granted bioRxiv a license to display the preprint in perpetuity. It is made available under aCC-BY 4.0 International license.

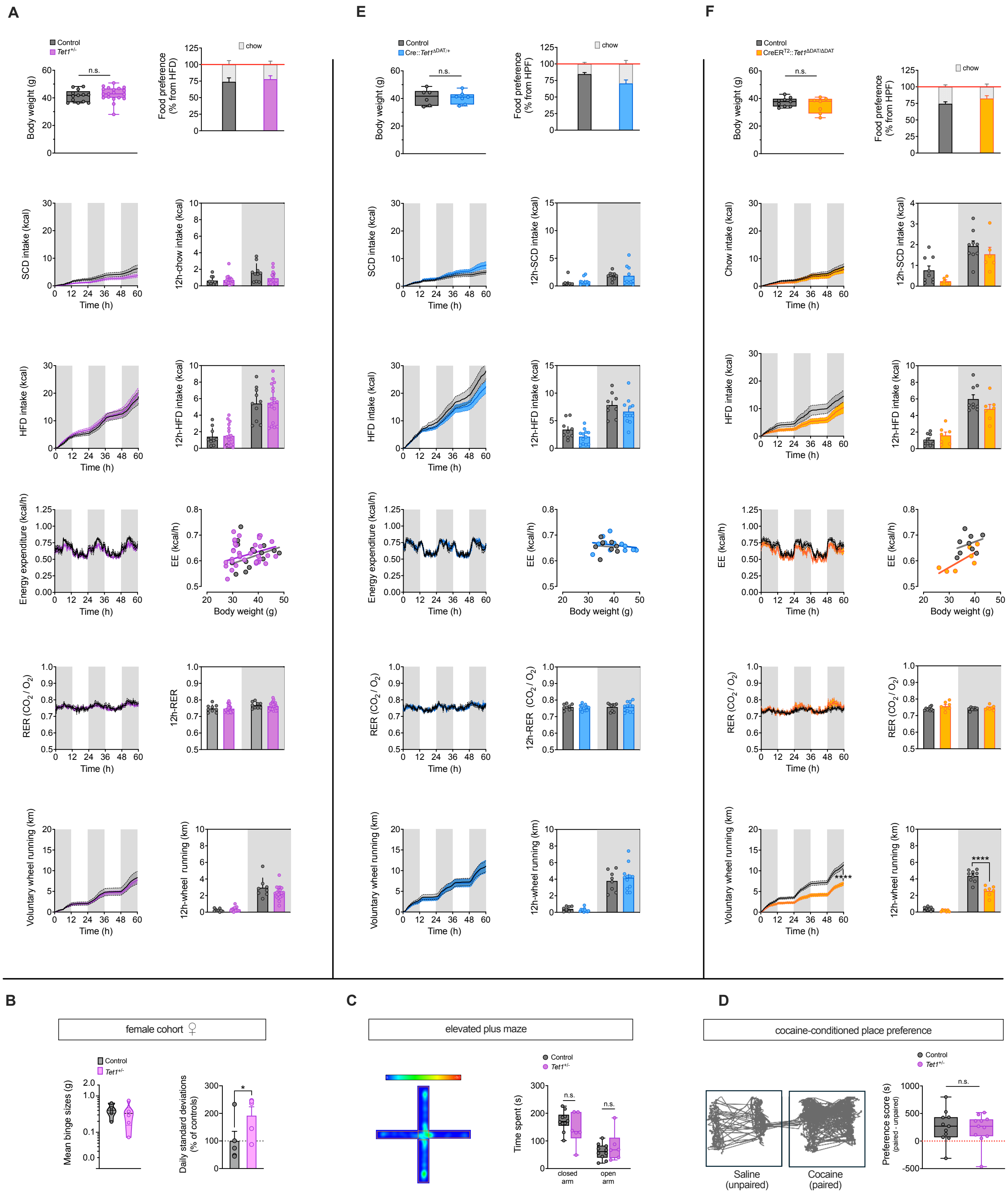

Supplementary Figure 3. Related to Figure 3. Heterozygous loss of *Tet1* triggers heterogeneity in binge-eating vulnerability. **(A)** In-depth metabolic phenotyping of male *Tet1*$^{+/-}$ mice. $n = 10$–22 mice. **(B)** Mean binge sizes of *Tet1*$^{+/-}$ female mice and female littermate controls (left panel), plotted on a Log$_{10}$ scale (shaded area: normal binge response). Data are presented as violin plots with median and quartiles. $n = 6$–7 mice. Standard deviation of daily binge sizes after Log$_{10}$ transformation relative to respective controls (right panel). Data are presented as mean $\pm$ SEM of STDEV from Day 1–5. $*P < 0.05$ (one-tailed Student's t-test). **(C)** Elevated plus maze (EPM) performed on male *Tet1*$^{+/-}$ mice and controls with representative movement tracking (left panel) and corresponding quantification (right panel). Data are presented as median $\pm$ minimum and maximum. $n = 6$–10 mice. **(D)** Cocaine-conditioned place preference performed on male *Tet1*$^{+/-}$ mice with representative movement tracking (left panel) and corresponding quantification (right panel). Data are presented as median $\pm$ minimum and maximum. $n = 11$ mice. **(E)** In-depth metabolic phenotyping of male Cre::*Tet1*$^{\Delta DAT/+}$. $n = 9$–12 mice. **(F)** In-depth metabolic phenotyping of male iCreER^{T2}::*Tet1*$^{\Delta DAT/\Delta DAT}$. $n = 7$–9 mice.

bioRxiv preprint doi: https://doi.org/10.64898/2026.03.14.711800; this version posted March 19, 2026. The copyright holder for this preprint (which was not certified by peer review) is the author/funder, who has granted bioRxiv a license to display the preprint in perpetuity. It is made available under aCC-BY 4.0 International license.

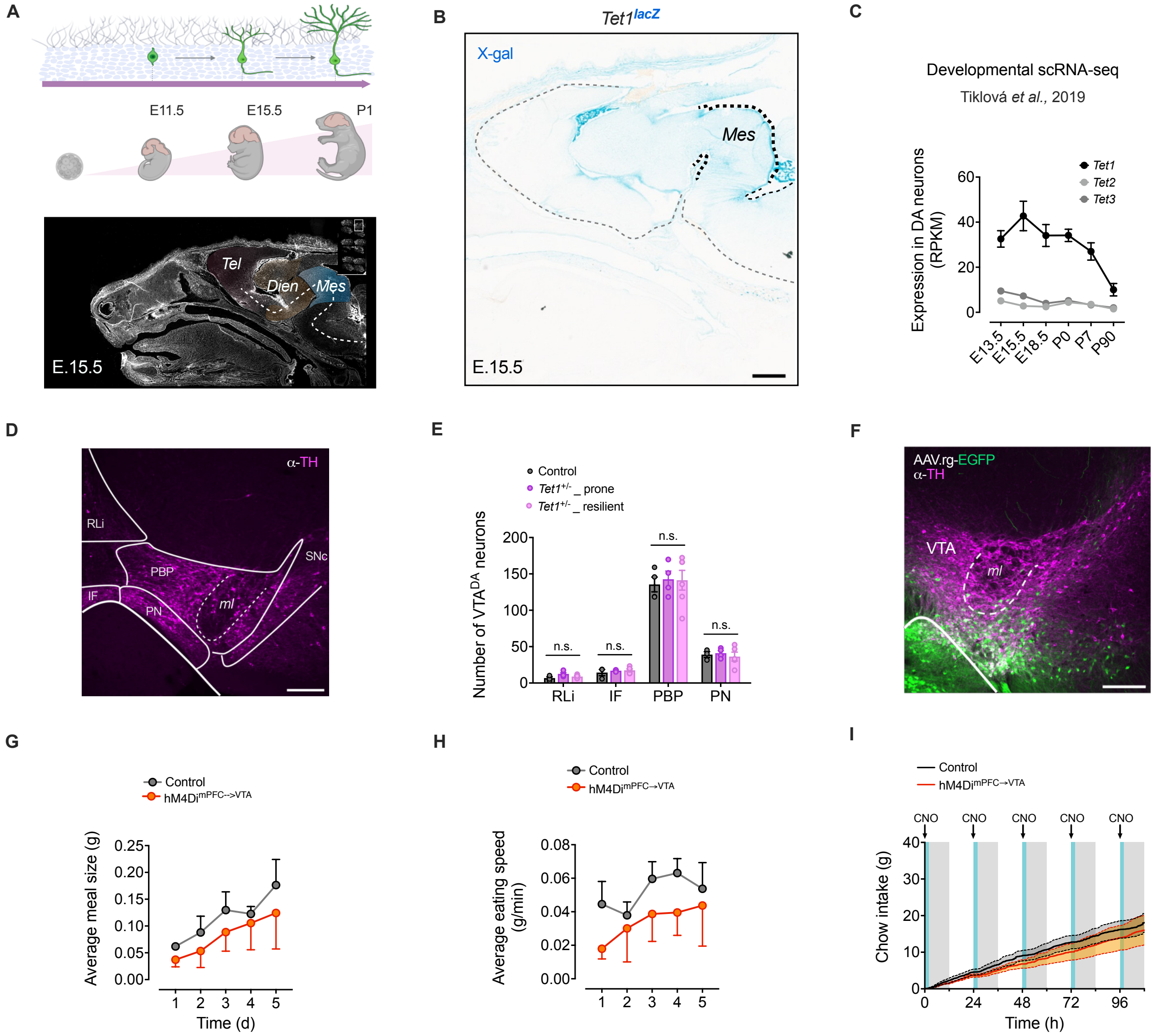

Supplementary Figure 4. Related to Figure 4. Reduced mPFC^{PL}$\rightarrow$ VTA inputs confer binge-eating resilience in *Tet1*$^{+/-}$ mice. (A) Sagittal overview of embryo head (E15.5) with pseudocolored brain regions (Telencephalon: red; Diencephalon: yellow; Mesencephalon: blue). *Created in BioRender.com.* **(B)** *Tet1*^{LacZ} reporter mouse embryo (E15.5) after X-gal staining (Mesencephalon highlighted). Scale bar: 500 µm. **(C)** Reanalysis of developmental scRNA-seq[58] showing expression levels of *Tet1*, *–2* and *–3* at different embryonic stages of development. **(D)** Micrograph showing VTA^{DA} neurons (magenta; TH^{+}) across different VTA subregions of an adult male mouse. **(E)** Quantification of VTA^{DA} neuron counts per VTA subregion. **(F)** Micrograph of injection site showing VTA^{DA} neurons (magenta; TH) and EGFP^{+} interneurons and fibers. **(G)** Average meal size per day of hM4Di$^{\text{mPFC-VTA}}$ mice relative to control mice. Data are presented as mean ± SEM. $n = 4$–7 mice. **(H)** Average eating speed per day of hM4Di$^{\text{mPFC-VTA}}$ mice relative to control mice. Data are presented as mean ± SEM. $n = 4$–7 mice. **(I)** Cumulative chow intake of hM4Di$^{\text{mPFC-VTA}}$ mice relative to control mice. Time window with limited HFD access (shaded) and CNO injection (1 mg/kg BW; i.p.; arrow) are indicated. Data are presented as mean ± SEM. $n = 4$–7 mice.

bioRxiv preprint doi: https://doi.org/10.64898/2026.03.14.711800; this version posted March 19, 2026. The copyright holder for this preprint (which was not certified by peer review) is the author/funder, who has granted bioRxiv a license to display the preprint in perpetuity. It is made available under aCC-BY 4.0 International license.

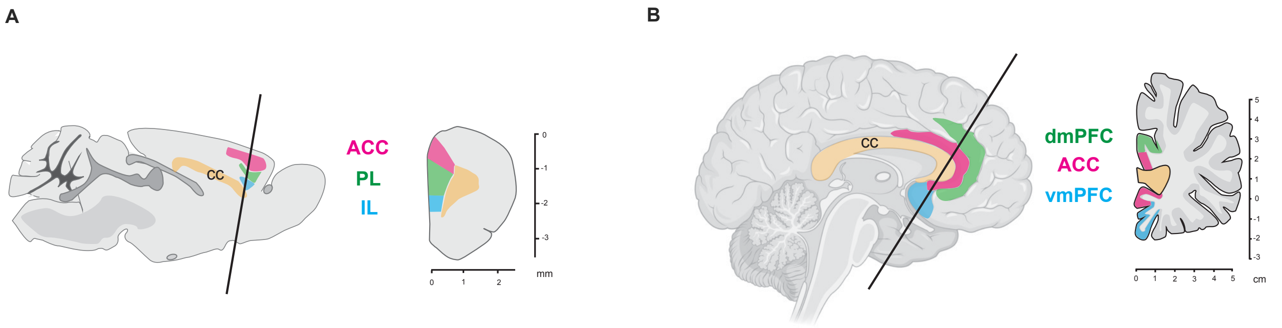

Supplementary Figure 5. Related to Figure 5. EGR1-guided TET1 reactivation in VTA^{DA} neurons restores binge-eating susceptibility. **(A)** Motif analyses on eBED-induced DMRs showing significant enrichment of indicated transcription factors, including EGR1. Adjusted p-values from one-tailed Fisher's exact test, followed by Bonferroni correction. **(B)** Quantification of c-Fos immunoreactivity punctae in the VTA and SNc at 1 h, 2 h or 4 h after the last of five binge-eating episodes versus chow-fed control mice. Data are presented as mean $\pm$ SEM. n.s. = not significant. $n = 4$–7 mice (one-way ANOVA). **(C)** Schematic illustration of EGR1-TET1.CD fusion protein. ZF: zinc finger domain, Cys-rich: cystine-rich domain, DSBH: double-stranded β-helix domain.

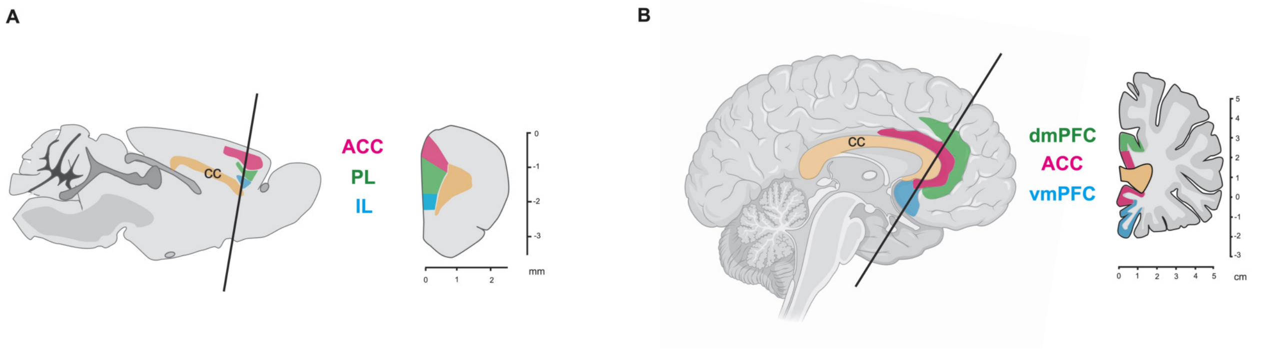

Supplementary Figure 6. Related to Figure 6. Human *TET1* methylation status mediates binge eating and dmPFC brain activation during reward processing. **(A)** Illustration of a mouse brain in sagittal (left) and coronal (right) orientation highlighting medial prefrontal cortex (mPFC) subregions. ACC: anterior cingulate (Cg1) mPFC, PL: prelimbic mPFC, IL: infralimbic mPFC. *Created in BioRender.com.* **(B)** Illustration of a human brain in sagittal (left) and coronal (right) orientation highlighting medial prefrontal cortex (mPFC) subregions. dmPFC: dorsomedial PFC, ACC: anterior cingulate mPFC, vmPFC: ventromedial PFC.

Homologous regions are color-coded (green: PL $\leftrightarrow$ dmPFC; red: ACC; blue: IL $\leftrightarrow$ vmPFC).
